# Sishen Pill inhibits intestinal inflammation in diarrhea mice via regulating kidney-intestinal bacteria-metabolic pathway

**DOI:** 10.3389/fphar.2024.1360589

**Published:** 2024-06-10

**Authors:** Xiaoya Li, Bo Qiao, Yueying Wu, Na Deng, Jiali Yuan, Zhoujin Tan

**Affiliations:** ^1^ School of Traditional Chinese Medicine, Hunan University of Chinese Medicine, Changsha, Hunan, China; ^2^ Hunan Key Laboratory of Traditional Chinese Medicine Prescription and Syndromes Translational Medicine, Changsha, Hunan, China; ^3^ College of Basic Medicine, Yunnan University of Chinese Medicine, Kunming, Yunnan, China; ^4^ Yunnan Provincal Key Laboratory of Chronic Disease Prevention and Treatment of Integrated Traditional Chinese and Western Medicine, Yunnan University of Chinese Medicine, Kunming, Yunnan, China

**Keywords:** Sishen Pill, diarrhea, characteristic bacteria, short-chain fatty acids, intestinal inflammatory, kidney function

## Abstract

**Background:**

Sishen Pill (SSP) has good efficacy in diarrhea with deficiency kidney-yang syndrome (DKYS), but the mechanism of efficacy involving intestinal microecology has not been elucidated.

**Objective:**

This study investigated the mechanism of SSP in regulating intestinal microecology in diarrhea with DKYS.

**Methods:**

Adenine combined with *Folium sennae* was used to construct a mouse model of diarrhea with DKYS and administered with SSP. The behavioral changes and characteristics of gut content microbiota and short-chain fatty acids (SCFAs) of mice were analyzed to explore the potential association between the characteristic bacteria, SCFAs, intestinal inflammatory and kidney function-related indicators.

**Results:**

After SSP intervention, the body weight and anal temperature of diarrhea with DKYS gradually recovered and approached the normal level. *Lactobacillus johnsonii* was significantly enriched, and propionic, butyric, isobutyric and isovaleric acids were elevated. Serum creatinine (Cr), interleukin-6 (IL-6) and tumour necrosis factor-α (TNF-α) levels of the mice were reduced, while serum blood urea nitrogen (BUN) and secretory immunoglobulin A (sIgA) in the colonic tissues were increased. Moreover, there were correlations between *L. johnsonii*, SCFAs, intestinal inflammatory, and kidney function.

**Conclusion:**

SSP might suppress the intestinal inflammation by regulating the “*L. johnsonii*-propionic acid” pathway, thus achieving the effect of treating diarrhea with DKYS.

## Introduction

Diarrhea is a common and frequent disease worldwide, with differences in Traditional Chinese medicine (TCM) syndromes due to its causes, pathogenesis and clinical manifestations (Li and Tan, 2020). As one of the common TCM symptoms of diarrhea, deficiency kidney-yang syndrome (DKYS) is still challenging to study on its pathogenesis due to its complex pathogenic factors (Li et al., 2020). Therefore, it is of great practical importance to investigate the mechanisms of diarrhea with DKYS in order to find effective diagnostic and therapeutic measures.

To date, with the popularization of high-throughput sequencing technology for gut microbiota, it is gradually recognized that alterations in the structure and function of gut microbiota are closely associated with diseases of the gastrointestinal system, cardiovascular system and urinary system, among others (Hobby et al., 2019; Verhaar et al., 2020; Xiao et al., 2021). Studies have pointed out that certain doses of adenine impaired kidney function to a certain extent and also affect the body’s energy metabolism (Jia et al., 2016). Dried leaflets of *Cassia senna* L. (Fabaceae) (*Folium Sennae*) is a bitter-cold laxative, and mice with diarrhea symptoms and caused gut microbiota disorders after the *Folium Sennae* modeling (Zhang et al., 2020a). Our preliminary research showed that adenine combined with *Folium Sennae* successfully replicated a diarrhea mouse model with DKYS (Li et al., 2022a; Zhou et al., 2024). Additionally, there was an imbalance of gut microbiota in diarrhea mice with DKYS (Zhu et al., 2016; Li et al., 2022b). Of course, SCFAs are also involved in the development of diarrhea with DYKS. Acetic, propionic, butyric, valeric, isobutyric and isovaleric acids in the gut contents of diarrhea mice with DYKS were significantly lower than those in the control group, suggesting that SCFAs were markedly inhibited by diarrhea with DYKS (Li et al., 2022b). Globally, gut microbiota and SCFAs has potential application in the diagnosis and risk assessment of diarrhea with DKYS.

Sishen Pill (SSP), as a classic formula for treating diarrhea with DKYS, is composed of six Chinese herbs, namely, *Psoralea corylifolia* L, *Myristica fragrans* Houtt, *Euodia rutaecarpa* (Juss.) Benth, *Schisandra chinensis* (Turcz.) Baill, *Ziziphus jujuba* Mill, *Zingiber officinale* Roscoe, which have the function of warming the kidney and spleen, consolidating the gut and relieving diarrhea (Li, 2012). It is often used clinically in the treatment of irritable bowel syndrome, ulcerative colitis, functional diarrhea, etc (Zhu et al., 2016; Chen et al., 2020; Liu et al., 2020; Li et al., 2022c; Li et al., 2022d; Ge et al., 2022; Li et al., 2023). Zhou et al. pointed out that SSP markedly increase the level of sIgA in diarrhea rats with DKYS, promoted the repair of gut mucosa, maintained the integrity of gut mucosa and other effects (Zhou, 2007). Liu et al. revealed that SSP reduced the number of Proteobacteria and *Mycoplasma* and increased *Clostridium* and other bacteria in the faeces of rats with diarrhoeal irritable bowel syndrome, suggesting that SSP could regulate the structure of gut microbiota to play a role in the treatment of diarrhoeal irritable bowel syndrome (Liu et al., 2019). Apparently, SSP has good efficacy in protecting the gut mucosa and regulating gut microbiota.

In this study, we used adenine combined with *Folium Sennae* to construct a mouse diarrhea model with DYKS and used SSP to intervene. Application of three-generation high-throughput sequencing technology combined with bioinformatics to uncover the interactions between characteristic bacterium at the species level and relevant environmental factors, and to elucidate the gut microecological mechanism of SSP intervention in diarrhea with DYKS. This study will provide a breakthrough for the study of the pharmacodynamic mechanism of SSP in the intervention of diarrhea with DYKS, and also provide an important reference for the intervention of TCM to optimize the therapeutic effect of diseases by regulating gut microorganisms.

## Materials and methods

### Drugs

Adenine (Changsha Yaer Biology Co., LTD, Changsha, China, number: EZ2811A135). *Folium Sennae* (Anhui Puren Traditional Chinese Medicine Yinpian Co. LTD, Haozhou, Anhui, number: 2005302). SSP composition: *P. corylifolia* (No: HY21012201), *M*. *fragrans* (No: Xiang 20160111), *E*. *rutaecarpa* (No: 2020082804), *S*. *chinensis* (No: HY21020304), *Z*. *jujuba* (No: 2103120082), *Z*. *officinale* (No: 170903). All the Chinese medicine tablets are provided by Hunan Junhao Chinese Medicine Tablets Science and Trade Co., LTD. Adenine suspension preparation (Xiao et al., 2016): adenine was prepared in sterile water to a concentration of 5 mg/mL in proportion to the concentration of the suspension and was prepared daily, as needed. *Folium Sennae* decoction preparation (Liu et al., 2019): we placed *Folium Sennae* in a container with the appropriate amount of water for 30 min. Then, we poured off the water, added 5 times the amount of herbs to the container and boiled for 30 min. We filtered out the liquid by laying sterile gauze flat in a funnel. The filtered drugs were then added to an appropriate amount of water and the decoction was continued by boiling for 15 min. The two decoctions were mixed and then boiled for 15 min. The decoction was concentrated to a concentration of 1 g/mL of raw herbs and stored in a refrigerator at 4 °C. SSP decoction preparation (Li et al., 2021): The preparation method was the same as *Folium Sennae* decoction preparation. Finally, the decoction was concentrated to a concentration of 0.29 g/mL of raw herbs and stored in a refrigerator at 4°C.

### Reagents

Interleukin-6 (IL-6) ELISA Kit (Jiangsu Jingmei Biotechnology Co., LTD., No. JM-02446M1). Tumor necrosis factor-α (TNF-α) ELISA Kit (Jiangsu Jingmei Biotechnology Co., LTD., No. JM-02415M1). Secretory immunoglobulin A (sIgA) ELISA Kit (Jiangsu Jingmei Biotechnology Co., LTD., No. JM-02723M1).

## Animals

Fifteen 4-week-old Kunming mice (male, 18–22 g) were supplied by the Slack Jingda Experimental Animal Co, Ltd (SCXK [Xiang] 2016–0002). Mice were housed in a controlled environment with free access to food and water. The room temperature was maintained at 23°C–25°C with a 12-h light/dark cycle. The animal experiments were approved by the Animal Ethics and Welfare Committee of Hunan University of Chinese Medicine (No. LLBH-202106120002). To exclude the effect of gender on the gut microbiota of mice, only male mice were used in this study (Wu et al., 2022).

### Experimental design

Modeling stage: Fifteen mice were randomly divided into five in the control (CZ) group and ten in the model (CX) group after 7 days of adaptive feeding. After modification of the modeling method with reference to the literature (Xiao et al., 2016; Xiao et al., 2016; Zhang et al., 2020a), mice in the CX group were given adenine suspension by gavage, 50 mg/(kg.d), 0.4 mL/each, once/d for 14 days. From the eighth day onwards, the model group was gavaged *Folium Sennae* decoction, 10 g/(kg.d), 0.4 mL/each, once/d for 7 days. Mice in the CZ group were given an equal volume of sterile water by gavage once/d for 14 days. In our previous experiments, we have successfully established and verified the reliability of a mouse diarrhea model with DYKS using the same modeling method (Li et al., 2022d).

TCM intervention stage: At the end of modeling, the CX group was randomly divided into two groups, i.e., five mice in the SSP (CS) group and five mice in the CX group. According to the conversion method of “conversion of drug dosage between experimental animals and human” in (Methodology of pharmacological research on Chinese medicine) (Li et al., 2021), the equivalent dose of Chinese medicine for gavage in the CS group of mice was calculated as 5 g/(kg.d), 0.35 mL/each, 2 times/d for 7 days. The CZ and CX groups were gavaged an equal volume of sterile water, 2 times/d for 7 days.

### Sample collection

Blood sample collection: At the end of the experiment, orbital blood was collected from all mice under aseptic conditions. Blood samples were collected for blood biochemistry and Enzyme-linked immunosorbent assay (ELISA).

Kidney sample collection: Kidney tissue was taken from mice under aseptic conditions. The connective tissue was removed from the surface of the kidneys and fixed in 4% paraformaldehyde solution for subsequent hematoxylin and eosin (H&E) staining.

Colonic sample collection: Colonic tissues were taken from mice under aseptic conditions. The contents of the colonic tissues were washed clean with sterile water. The cleaned colonic tissues were then placed in sterile EP tubes, labelled and stored at −80°C for subsequent ELISA.

Small intestine contents sample collection: Small intestine was taken from mice under aseptic conditions. We collected samples of contents from small intestinal tissue. The content samples from each mouse were placed individually in sterile EP tubes, labelled and stored in a refrigerator at −80°C for 16S rRNA gene subsequent high-throughput sequencing and gas chromatography-mass spectrometry (GC-MS).

### Ultra-high performance liquid chromatograph (UPLC) of SSP

The chemical components of the SSP extracts were confirmed by UPLC fingerprinting analysis. The samples were identified using an Thermo Fisher Scientific UPLC (Vanquish, United States) and a Q-Exactive™. Briefly, 300 μL of SSP supernatant was added to 1,000 μL of extraction solution (methanol: water = 4:1, internal standard concentration of 10 μg/mL). Vortex for 30 s and then sonicate for 5 min in an ice water bath. The sample was centrifuged at 4 °C for 15 min at 12,000 rpm (centrifugal force 13,800 (×*g*), radius 8.6 cm) after resting at −40°C for 1 h. The supernatant was removed and passed through a 0.22 μM membrane into a sample vial for detection in the machine. The samples were processed on a Waters UPLC BEH C18 column (1.7 μm * 2.1 * 100 mm) with an injection volume of 5 μL. See Table 1 for details.

**TABLE 1:** Mobile phase condition of chormatographic separation.

Times (min)	Flow rate (μL/min)	Mobile phase A (%)	Mobile phase B (%)
0	500	85	15
11	500	25	75
12	500	2	98
14	500	2	98
14.1	500	85	15
16	500	85	15

Mobile phase A comprising water. Mobile phase B comprising acetonitrile. 0.1% formic acid was added to both mobile phase A and B.

### Behavioural observations in mice

At the end of the experiment, the behavioral status of the mice in the three groups was observed. The indicators observed here included: the mental state of the mice, activity flexibility, hair color, fecal character and color, and anal cleanliness (Li et al., 2022e). Besides, the body weight and anal temperature of the mice were measured on days 1, 5, 9, 13, 15, 18 and 21 of the experiment.

### Pathological observation of kidney tissue in mice

At the end of the experiment, the kidney tissues fixed in 4% paraformaldehyde solution were removed, dehydrated in gradient ethanol, transparent in xylene, routinely paraffin embedded, sectioned and stained with H&E and then observed under a light microscope for histopathological changes.

### Blood creatinine (Cr) and urea nitrogen (BUN) assay in mice

The fully automated biochemical analyser was used to determine the Cr and BUN levels in mice. Firstly, the machine was switched on and warmed up to complete the calibration, quality control and sample setting serial numbers. Next, the calibrator, quality control and test samples were loaded according to the set serial numbers. Finally, the instrument was run to complete the calibration, quality control determination and sample testing.

### Detection of sIgA level in mice colonic tissue

The collected mice colon tissue was mashed with appropriate amount of saline at 3000 r, centrifuged for 10 min, and the supernatants were taken. Added 10 μL of supernatant and 40 μL of sample dilution to the sample wells as prompted by the ELISA instructions. Added 50 μL of different concentrations of standards to the standard wells. Then, added 100 μL of horseradish peroxidase HRP-labeled antibody to the standard and sample wells, respectively. The reaction wells were sealed with sealing film and incubated in a water bath for 60 min. After the incubation was completed, the slats were removed and the liquid was poured off, patted dry on absorbent paper, each well was filled with pre-configured washing solution, left for a few minutes and poured off, and repeated 5 times. After the incubation, 50 μL of substrate A and 50 μL of substrate B were added to each well and incubated in a water bath for 15 min 50 μL of termination solution was added to each well after the incubation. The OD value of each well was measured with an enzyme marker.

### Detection of serum IL-6 and TNF-α levels in mice

Blood samples were allowed to stand for 30 min at room temperature, centrifuged at 3,000 r/min for 10 min, serum was separated and assay samples were loaded into centrifuge tubes. The procedure for ELISA of IL-6 and TNF-α in serum was the same as that for sIgA in colon tissue described above.

### Gut content microbiota assay

Total DNA extraction and amplification, PCR amplification, recovery and purification of amplification products, fluorescence quantification of amplification products, and computer testing were used to detect the gut content microbiota of mice.

Primer design: forward primer 27F (5′-AGAGTTTGATCMTGGCTCAG-3′)

reverse primer 1492R (5′-GGACTACHVGGGTWTCTAAT-3′).

The prepared DNA libraries were sequenced on the PacBio Sequel platform. The sequencing was performed by Paiseno Biological Co., LTD.

Detection of the acetic acid, propionic acid, butyric acid, isobutyric acid, valeric acid and isovaleric acid in the gut content microbiota.

We detected SCFAs in the gut contents microbiota of mice by GC-MS. The GC-MS conditions are shown in Table 2. The above testing process was completed by Qingdao Yixin Co., LTD (Qingdao, China), and the above preparation procedures were performed by the GC-MS external standard method.

**TABLE 2:** GC-MS conditions.

Steps	Conditions
Column temperature requirement	100°C (5 min)-5°C/min-150°C (0 min)-30°C/min-240°C (30 min)
Flow rate requirements	1 mL/min
Shunt ratio	75:1
Carrier gas	Helium
Chromatographic column	TG WAX 30 m × 0.25 mm × 0.25 μm
Injector	240°C
Mass spectrometry EI source, bombardment voltage	70 eV
Single ion scan mode	Quantitative ion 63, 70
Ion source temperature	200°C
Connection line temperature	250°C

### Statistical analysis

SPSS 21.00 software was used for statistical analysis and data obtained from each group were expressed as mean ± standard deviation. If the data were consistent with normal distribution and homogeneity of variance, one-way analysis of variance was used for comparison between groups, and LSD method was used for pair comparison between groups; otherwise, Kruskal–Wallis H test was used. *p* < 0.05 was considered statistically significant. *p* < 0.01 was considered extremely statistically significant.

## Results

### The active components of SSP

**FIGURE 1:**
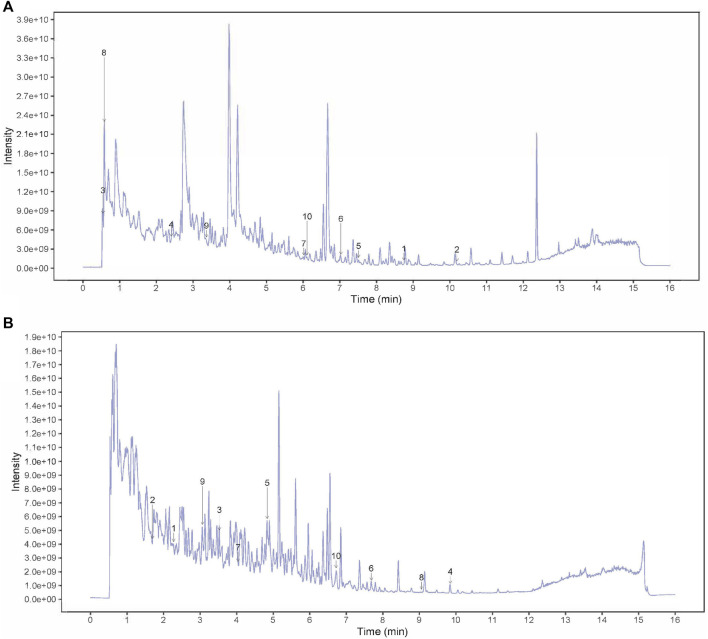
HPLC fingerprint chromatogram of SSP. **(A)** peak intensity chromatograms of SSP in positive mode. Numbers 1–10 in the figure represented Benzaldehyde, Biochanin A, Citric acid, Codeine, Dehydroevodiamine, Derricidin, Epimedokoreanin B, NICOTINAMIDE, OBLIQUIN, Phthalic anhydride. **(B)** peak intensity chromatograms of SSP in negative mode. Numbers 1–10 in the figure represented (2R,3S,4S,5R,6S)-2-(hydroxymethyl)-6-[4-(hydroxymethyl)-1-propan-2-ylcyclohex-3-en-1-yl]oxyoxane-3,4,5-triol, [(3aR,4S,6E,10Z,11aR)-10-(hydroxymethyl)-6-methyl-3-methylidene-2-oxo-3a,4,5,8,9,11a-hexahydrocyclodeca[b]furan-4-yl] (Z)-4-acetyloxy-2-(hydroxymethyl)but-2-enoate, [(9R,10R)-10-acetyloxy-8,8-dimethyl-2-oxo-9,10-dihydropyrano[2,3-f]chromen-9-yl] 2-methylbutanoate, 8-Geranyloxypsoralen, Adenine, Arylbenzofuran flavonoid base + 3O, 1MeO, 1Prenyl, Baicalein, Confertifoline, Daidzein, Demethoxycurcumin.

The fingerprint chromatogram of the SSP is shown in Figures 1A, B. There were 20 main peaks in the fingerprint of SSP, with the main peaks separated. Among these, seven kinds of components were detected including phenylpropanoids, alkaloid, flavonoids, coumarins and derivatives, terpenoids, miscellaneous, based on comparison with standard materials (Table 3).

**TABLE 3:** Identification of components of SSP.

Name	Formula	mzmed	RT (s)	Adduct	Peak area
(2R,3S,4S,5R,6S)-2-(hydroxymethyl)-6-[4-(hydroxymethyl)-1-propan-2-ylcyclohex-3-en-1-yl]oxyoxane-3,4,5-triol	C_16_H_28_O_7_	377.1821005	136.447	[M + FA]-	107070656.7
[(3aR,4S,6E,10Z,11aR)-10-(hydroxymethyl)-6-methyl-3-methylidene-2-oxo-3a,4,5,8,9,11a-hexahydrocyclodeca[b]furan-4-yl] (Z)-4-acetyloxy-2-(hydroxymethyl)but-2-enoate	C_22_H_28_O_8_	419.1709698	101.635	[M-H]-	17548560.49
[(9R,10R)-10-acetyloxy-8,8-dimethyl-2-oxo-9,10-dihydropyrano[2,3-f]chromen-9-yl] 2-methylbutanoate	C_21_H_24_O_7_	387.1445594	211.524	[M-H]-	394365745.6
8-Geranyloxypsoralen	C_21_H_22_O_4_	337.1438079	590.71	[M-H]-	220946443.3
Adenine	C_5_H_5_N_5_	134.0470878	290.082	[M-H]-	6503245.467
Arylbenzofuran flavonoid base + 3O, 1MeO, 1Prenyl	C_20_H_20_O_5_	339.1227214	461.059	[M-H]-	227580465.5
Baicalein	C_15_H_10_O_5_	269.0453506	242.352	[M-H]-	63461757.69
Confertifoline	C_15_H_22_O_2_	233.1541122	543.354	[M-H]-	9532757.167
Daidzein	C_15_H_10_O_4_	253.0503813	183.739	[M-H]-	581497795.3
Demethoxycurcumin	C_20_H_18_O_5_	337.1077325	403.169	[M-H]-	168202194.5
Benzaldehyde	C_7_H_6_O	107.0489833	524.789	[M + H]+	1427319.656
Biochanin A	C_16_H_12_O_5_	285.1690661	611.399	[M + H]+	1518437.506
Citric acid	C_6_H_8_O_7_	215.0160204	32.6875	[M + Na]+	14686871.35
Codeine	C_18_H_21_NO_3_	300.1590339	144.321	[M + H]+	17457057.14
Dehydroevodiamine	C_19_H_15_N_3_O	302.1283775	450.671	[M + H]+	37546366.02
Derricidin	C_20_H_20_O_3_	309.1460561	421.7585	[M + H]+	18883073.18
Epimedokoreanin B	C_25_H_26_O_6_	423.1807168	361.043	[M + H]+	14340426.7
NICOTINAMIDE	C_6_H_6_N_2_O	123.0551277	34.6752	[M + H]+	45625826.71
OBLIQUIN	C_14_H_12_O_4_	245.0809735	201.937	[M + H]+	13101520.82
Phthalic anhydride	C_8_H_4_O_3_	149.0235937	366.293	[M + H]+	2965704.824

### SSP induced behavioral change in diarrhea mice with DYKS

**FIGURE 2:**
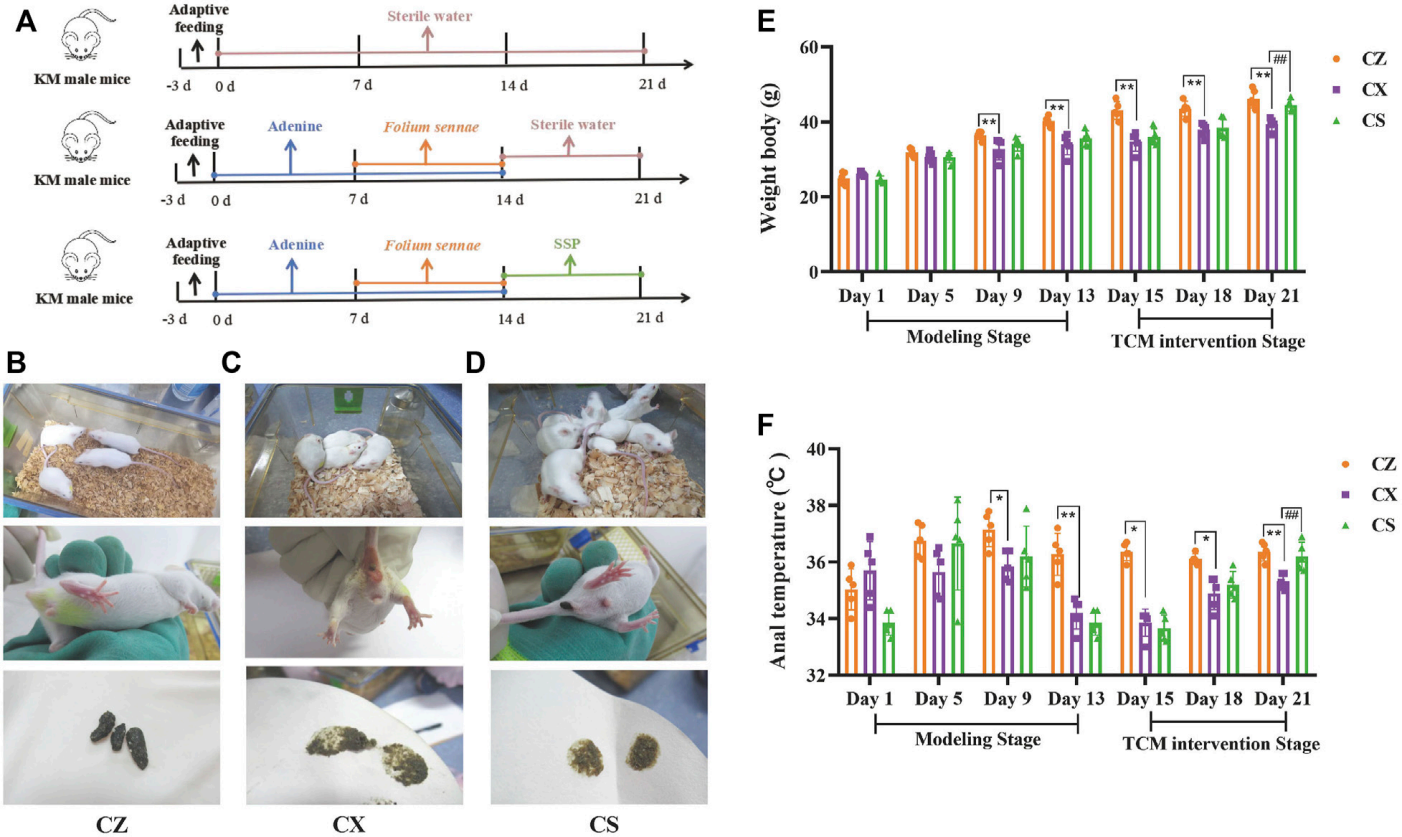
Behavioral changes in the mice. **(A)** Experimental flow chart. **(B)** Mental status, perianal condition and fecal characteristics of the mice in the CZ group. **(C)** Mental status, perianal condition and fecal characteristics of the mice in the CX group. **(D)** Mental status, perianal condition and fecal characteristics of the mice in the CS group. **(E)** Body weight of the mice. **(F)** Anal temperature of the mice. CZ, control group (*n* = 5); CX, model group (*n* = 5); CS, SSP group (*n* = 5). SSP: Sishen Pill; SCFAs: short-chain fatty acids. The values were expressed as mean ± standard deviation. Compared to CZ group, ^*^ *p* < 0.05, ^**^ *p* < 0.01. Compared to CX group, ^##^ *p* < 0.01.

The specific experimental procedure was shown in Figure 2A. Compared with the CZ group, the mice in the CX group were not in good spirits, sleepy and lazy, unresponsive, with arched backs, curled up in piles, sparse and lustrous fur, damp bedding, soft stools that stuck to the bedding and dirt around the anus. The mental state of mice in the CS group recovered somewhat, with flexible activities, improved fur gloss, reduced lethargy, lazy movement and clumping, and improved fecal laxity and perianal pollution (Figures 2B–D).

During the modeling stage, the body weight of mice in both the CX and CS groups were lower than those in the CZ group as the modeling days increased, with the body weights of mice in the CX group being significantly lower than those in the CZ group on days 9 and 13 of modeling (*p* < 0.01; *p* < 0.01). As the days of SSP intervention increased, the body weight of mice in the CS group gradually increased. At day 21, the body weight of mice in the CS group was markedly higher than that of the CX group (*p* < 0.01) and gradually approached that of the CZ group (Figure 2E).

As shown in Figure 2F, the anal temperature of both CX and CS mice was lower than that of the CZ group during the modeling stage, and was considerably lower in the CX group than in the CZ group on days 9 and 13 of modeling (*p* < 0.05; *p* < 0.01). With the increase of SSP intervention, the anal temperature of CS group mice increased gradually. On the 21st day of the experiment, anal temperature in CS group was significantly higher than that in CX group (*p* < 0.01), and gradually approached that in CZ group.

It could be seen that SSP altered the symptoms and signs and restored the body weight and anal temperature of diarrhea mice with DYKS.

### SSP changed the kidney function in diarrhea mice with DYKS

**FIGURE 3:**
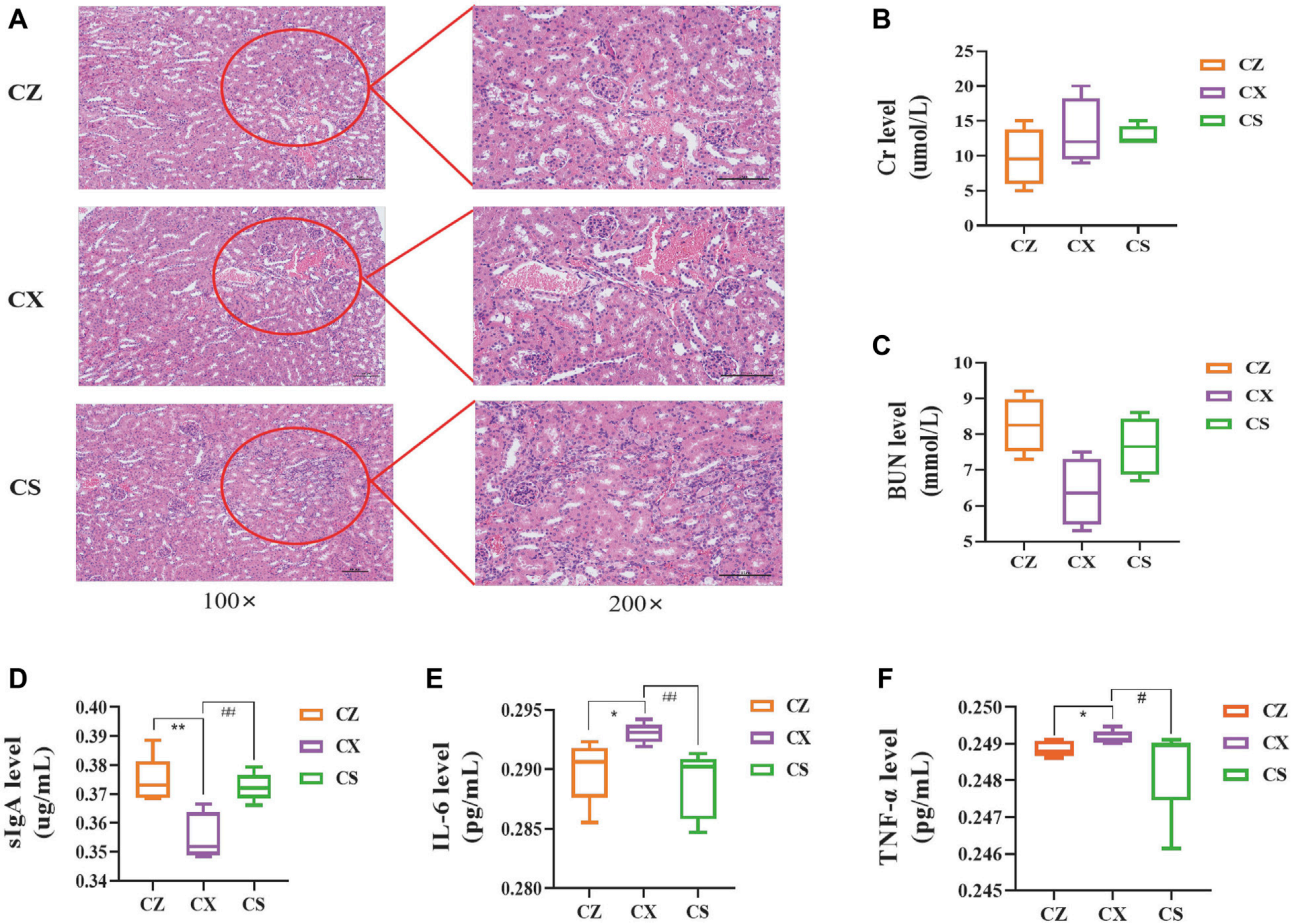
Indicators related to the kidney function and intestinal inflammatory response. **(A)** HE staining of the kidney. **(B)** Cr level. **(C)** BUN level. **(D)** sIgA level. **(E)** IL-6 level. **(F)** TNF-α level. CZ, control group (n = 5); CX, model group (*n* = 5); CS, SSP group (*n* = 5). Cr: creatinine; BUN: blood urea nitrogen; IL-6: interleukin-6; TNF-α: tumour necrosis factor-α; sIgA: secretory immunoglobulin **(A)**. The values were expressed as mean ± standard deviation. Compared to CZ group, ^*^ *p* < 0.05, ^**^ *p* < 0.01. Compared to CX group, ^#^ *p* < 0.05, ^##^ *p* < 0.01.

As depicted in Figure 3A, compared with the CZ group, the mice in the CX group presented a certain degree of pathological damage, with marked aggregation of inflammatory cells, edema and congestion in the renal interstitium, and tubular dilatation. However, the above injury was dramatically reduced in the CS group. After SSP intervention, serum Cr level of mice showed a decreasing trend compared with that in the CX group (*p* > 0.05), whereas BUN level presented a tendency to increase (*p* > 0.05) (Figures 3B, C), demonstrating that SSP caused changes in the kidney function of diarrhea mice with DYKS.

### SSP inhibited the intestinal inflammatory response in diarrhea mice with DYKS

Compared with the CZ group, sIgA levels in colonic tissues of mice in the CX group were notably lower (*p* < 0.01) and serum levels of IL-6 and TNF-α were significantly higher (*p* < 0.05; *p* < 0.05). Yet, sIgA level were markedly higher in the CS group of mice compared to the CX group (*p* < 0.01) (Figure 3D), and levels of IL-6 and TNF-α were enormously lower (*p* < 0.01; *p* < 0.05) (Figures 3E, F). So, SSP considerably inhibited the occurrence of intestinal inflammatory response in diarrhea mice with DYKS.

### SSP altered the gut content microbiota in diarrhea mice with DYKS

#### Quality assessment of sequencing data

**FIGURE 4:**
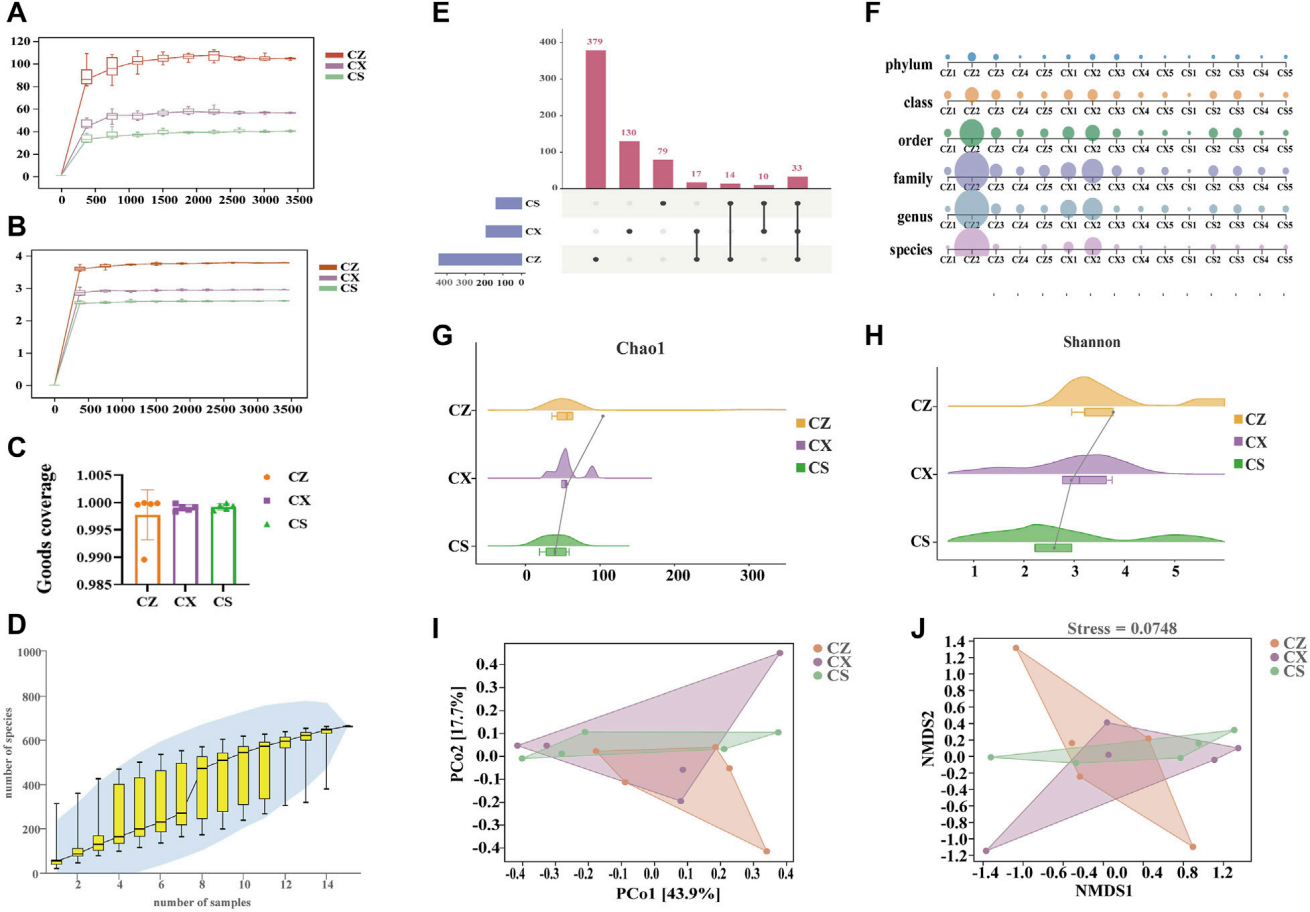
Quality assessment of sequencing data analysis of the OTU and diversity of the gut content microbiota. **(A)** Chao1 dilution curve. **(B)** Shannon dilution curve. **(C)** Goods coverage index. **(D)** Species accumulation curves. **(E)** Upset diagram. **(F)** Multiaxial bubble diagram. **(G)** Chao1 index. **(H)** Shannon index. **(I)** PCoA analysis. **(J)** NMDS analysis. CZ, control group (*n* = 5); CX, model group (*n* = 5); CS, SSP group (*n* = 5). PCoA: Principal Coordinate analysis; NMDS: non-metric multidimensional scaling.

As seen in the results, the sequenced sequences of all samples showed an inflection point around 500, and as the sequencing depth increases, the curve flattens out and reaches a plateau (Figures 4A, B). Moreover, the Goods coverage index of the samples within the same group was basically above 99% (Figure 4C), pointing out that the coverage of the samples within the group was good and no outliers were present. As illustrated in Figure 4D, the curve flattens out as the sample size increases, suggesting that the total number of OTUs barely increases as new samples were added. The above displayed that the sampling for this study was adequate to meet the needs of the study. In brief, it was proved that the experimental data meet the needs of the experimental design and downstream analysis.

### SSP adjusted the diversity of gut content microbiota in diarrhea mice with DYKS

There were 412 OTUs and 379 unique OTUs in the CZ group, 163 OTUs and 130 unique OTUs in the CX group, 112 OTUs and 79 unique OTUs in the CS group, and a total of 33 OTUs in the three groups (Figure 4E). SSP intervention markedly altered the changes in gut content microbiota in mice at six taxonomic levels (Figure 4F). Also, both the Chao1 and Shannon indexes of gut content microbiota in the CX group were reduced compared to those in the CZ group, and they were both reduced in the CS group compared to the CX group (Figures 4G, H). In the PCoA analysis (Figure 4I), the contribution of the horizontal coordinate PCo1 was 43.9% and the PCo2 was 17.7%. In the NMDS analysis, we found (Figure 4J) that the three groups of mice gut content communities had different structural distribution characteristics with a stress value of 0.0784, indicating that the grouping was reasonable. Altogether, SSP adjusted the diversity of gut content microbiota in diarrhea mice with DYKS.

### SSP reshaped the dominant bacteria and enriched the characteristic bacteria in diarrhea mice with DYKS

**FIGURE 5:**
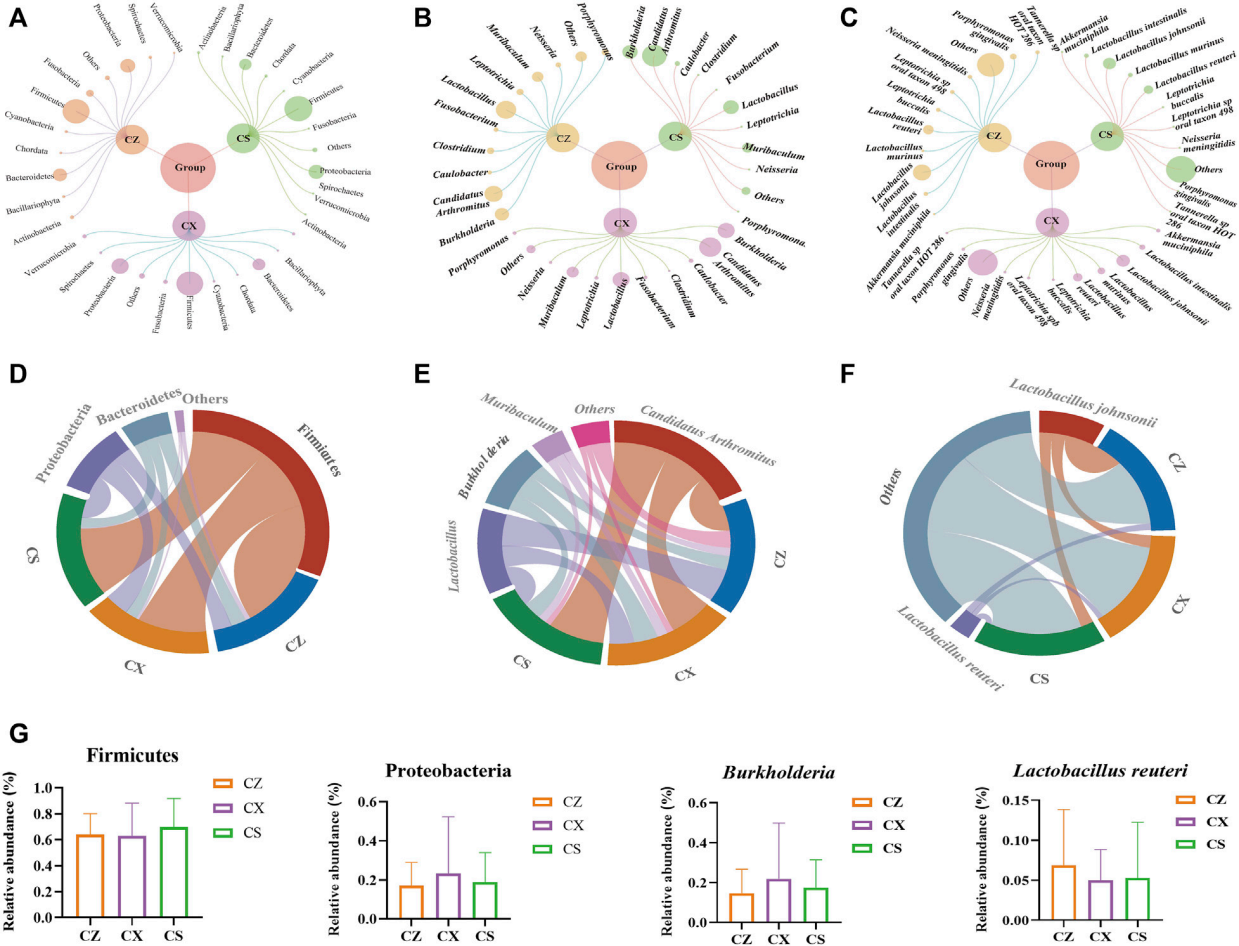
Analysis of the dominant bacteria of the gut contents in the mice. **(A)** Relative abundance diagram of the phylum level. **(B)** Relative abundance diagram of the genus level. **(C)** Relative abundance diagram of the species level. **(D)** Chord chart of the phylum level. **(E)** Chord chart of the genus level. **(F)** Chord chart of the species level. **(G)** Phyla, genus, species with a changing tendency. CZ, control group (*n* = 5); CX, model group (*n* = 5); CS, SSP group (*n* = 5).

At the phylum level, Firmicutes, Proteobacteria and Bacteroidetes accounted for the larger proportion of the gut contents in the three groups (Figure 5A). We used a chord diagram to summarize the dominant bacteria with abundance greater than 1% (Figures 5D–F). After statistical analysis of the above dominant bacteria (Figure 5G), it was found that compared with CX group, *Candidatus Arthromitus*, *Lactobacillus*, *Burkholderia* and *Muribaculum* were the dominant genera in all three groups with greater than 1% abundance (Figure 5B). *Burkholderia* tended to decrease in the CS group compared to the CX group, but none of the differences were statistically significant (Figure 5G). Moreover, *Lactobacillus johnsonii* and *Lactobacillus reuteri* were the dominant species in all three groups with greater than 1% abundance (Figure 5C). *Lactobacillus reuteri* showed an increasing trend in the CS group compared to the CX group (Figure 5G). So, the composition of the dominant bacteria of diarrhea mice with DYKS changed at the phylum, genus and species level after SSP intervention.

**FIGURE 6:**
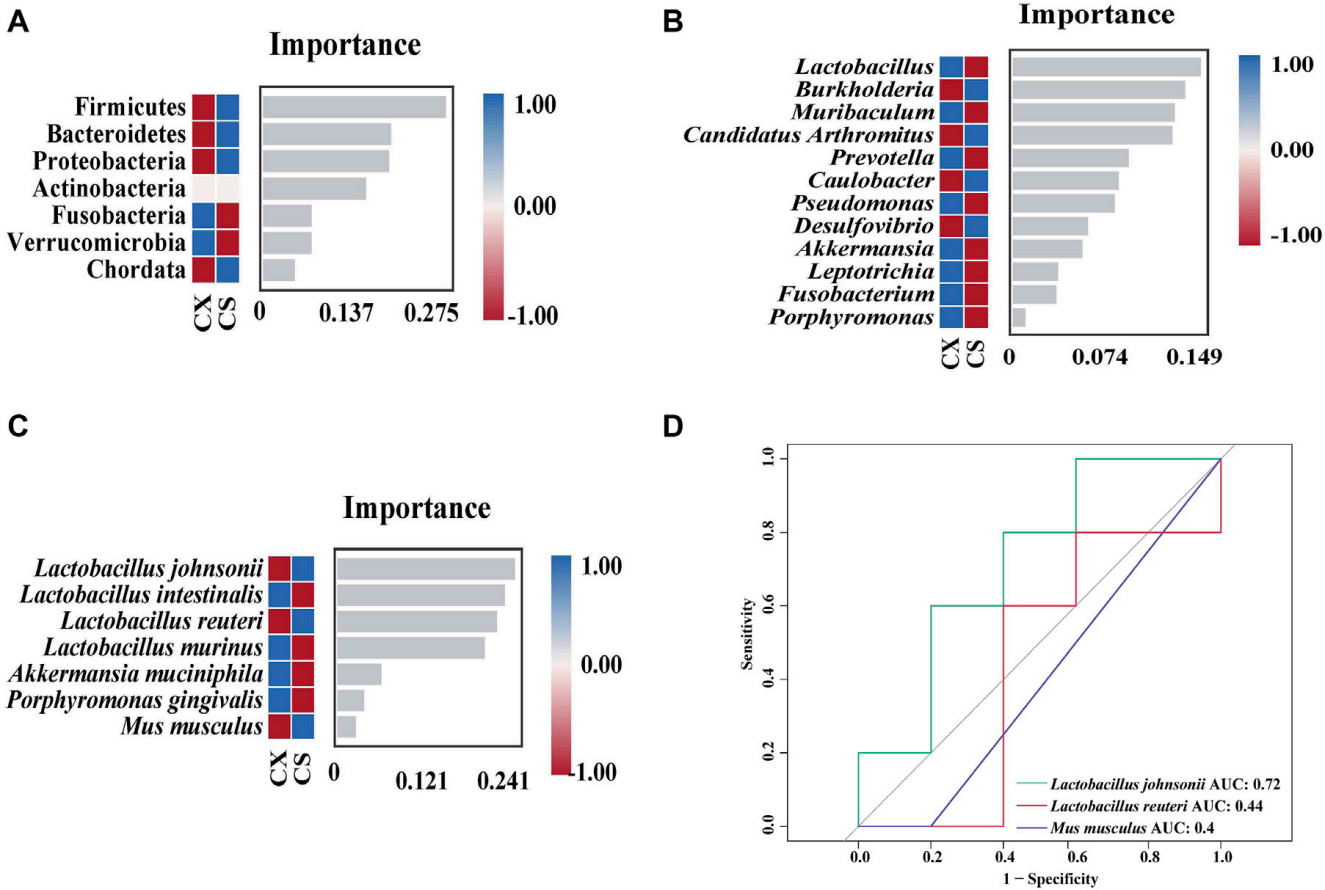
Analysis of the characteristic bacteria of the gut contents in the mice. **(A)** Random forest diagram of the phylum level. **(B)** Random forest diagram of the genus level. **(C)** Random forest diagram of the species level. **(D)** ROC of the species level. CZ, control group (*n* = 5); CX, model group (*n* = 5); CS, SSP group (*n* = 5). AUC: area under the curve.

At the phylum level, Firmicutes, Bacteroidetes, Proteobacteria and Chordata were identified as the key characteristic bacteria (Figure 6A). Four key characteristic bacteria were recognized at the genus level, including *Burkholderia*, *Candidatus Arthromitus* and *Caulobacter* (Figure 6B). *Lactobacillus johnsonii, L. reuteri* and *Mus musculus* were classified as key characteristic bacteria at the species level (Figure 6C). Subsequently, ROC curve analysis was performed on the three characteristic bacteria enriched at the species level in the CS group (Figure 6D). The area under the curve (AUC) was calculated to determine the value of the operating characteristic curve in predicting disease (Zhang et al., 2022). The results showed that *L. johnsonii* (AUC = 0.72) presented a large AUC value, indicating that *L. johnsonii* might be a potential biomarker for SSP in treating diarrhea with DYKS.

### SSP affected the function in diarrhea mice with DYKS

**FIGURE 7:**
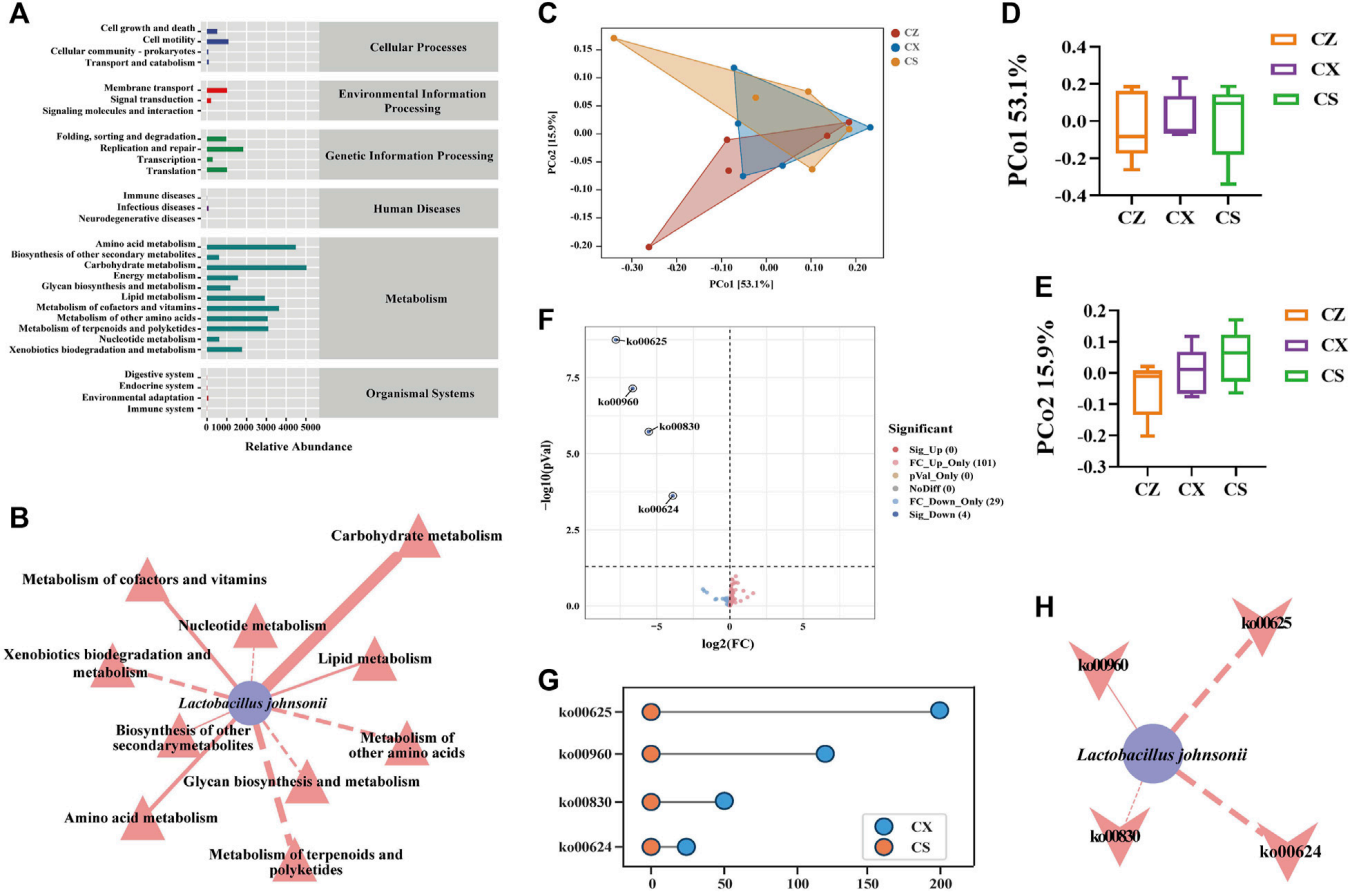
Functional analysis of the gut content microbiota in mice. **(A)** Predicted abundance of the KEGG function. **(B)** Network diagram of *Lactobacillus johnsonii* and metabolic function. **(C)** PCoA diagram of the KEGG functional units. **(D)** PCo1 analysis. **(E)** PCo2 analysis. **(F)** CX group vs. CS group volcano map. **(G)** CX group vs. CS group dumbbell diagram. **(H)** Network diagram of the *Lactobacillus johnsonii* and KEEG pathways. CZ, control group (*n* = 5); CX, model group (*n* = 5); CS, SSP group (*n* = 5). PCo1: Principal Coordinate 1; PCo2: Principal Coordinate 2.

Combined with KEGG functional cluster analysis (Figure 7A), it was found that the first-level functions of gut content microbiota were generally divided into six categories, and the second-level functions had a total of 29 seed functional categories, with a greater abundance of sub-functional classes under metabolism. We applied Cytoscape 3.7.2 to construct a “characteristic bacteria-metabolic function” interaction network to reflect the correlation between characteristic bacteria and metabolic function in the treatment of diarrhea with DYKS with SSP (Figure 7B). The characteristic bacteria *L. johnsonii* were significantly positively correlated with carbohydrate metabolism and negatively correlated with metabolism of terpenoids and polyketides. In the PCoA analysis, the contribution rate of PCo1 in horizontal coordinate was 53.1%, and that of PCo2 in vertical coordinate was 15.9% (Figures 7C–E). A total of 134 homologous genes were predicted, including four downregulated KOs with statistical differences (Figure 7F). Among them, pathways associated with the CS group included ko00625 (chloroalkane and chloroalkene degradation), ko00960 (tropane, piperidine and pyridine alkaloid biosynthesis), ko00830 (retinol metabolism) and ko00624 (polycyclic aromatic hydrocarbon degradation) (Figure 7G). Later, we conducted correlation analysis between *L. johnsonii* and the kyoto encyclopedia of genes and genomes (KEGG) pathway with significant differences to explore the assocation between the characteristic bacteria of gut content in mice after the intervention of SSP and the KEGG pathway (Figure 7H). *Lactobacillus johnsonii* was negatively correlated with ko00624 (polycyclic aromatic hydrocarbon degradation), ko00625 (chloroalkane and chloroalkene degradation) and ko00830 (retinol metabolism), whereas positively correlated with ko00960 (tropane, piperidine and pyridine alkaloid biosynthesis). Hence, the above metabolic pathway may be the main way for SSP to intervene in the change of gut content microbiota in diarrhea mice with DYKS.

### SSP caused changes of SCFAs in diarrhea mice with DYKS

**FIGURE 8:**
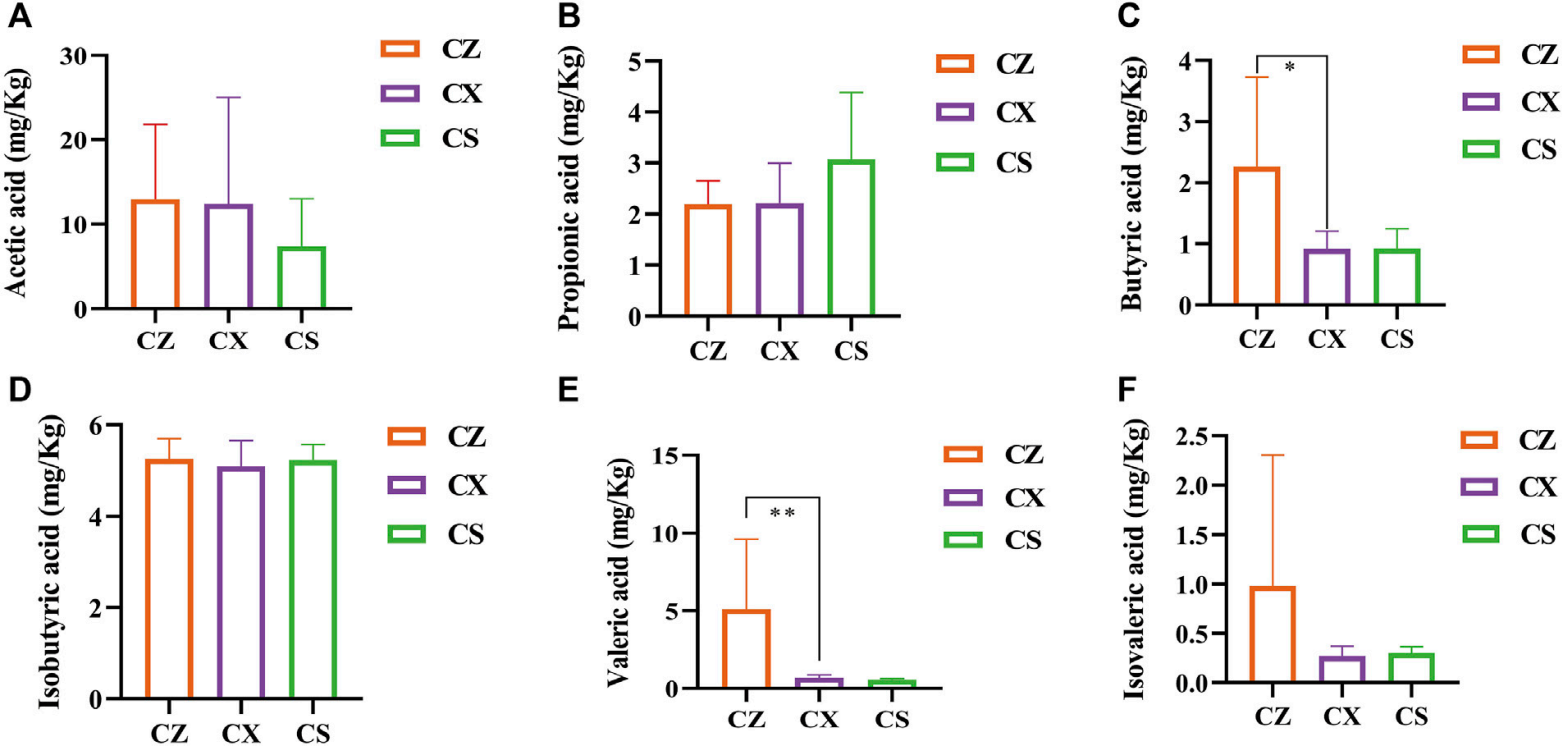
Analysis of SCFAs. **(A)** Acetic acid. **(B)** propionic acid. **(C)** Butyric acid. **(D)** Valeric acid. **(E)** Isobutyric acid. **(F)** Isovaleric acid. CZ, control group (*n* = 5); CX, model group (*n* = 5); CS, SSP group (*n* = 5). The values were expressed as mean ± standard deviation. Compared to CZ group, ^**^ *p* < 0.01.

Compared with the CX group, propionic acid, butyric acid, isobutyric acid and isovaleric acid were all increased in the gut contents of mice in the CS group (*p* > 0.05; *p* > 0.05; *p* > 0.05; *p* > 0.05), while acetic acid and valeric acid showed a decreasing trend (*p* > 0.05; *p* > 0.05) (Figures 8A–F). It was suggested that the intervention of SSP caused changes in SCFAs in the gut contents of diarrhea mice with DYKS.

### Correlation analysis

**FIGURE 9:**
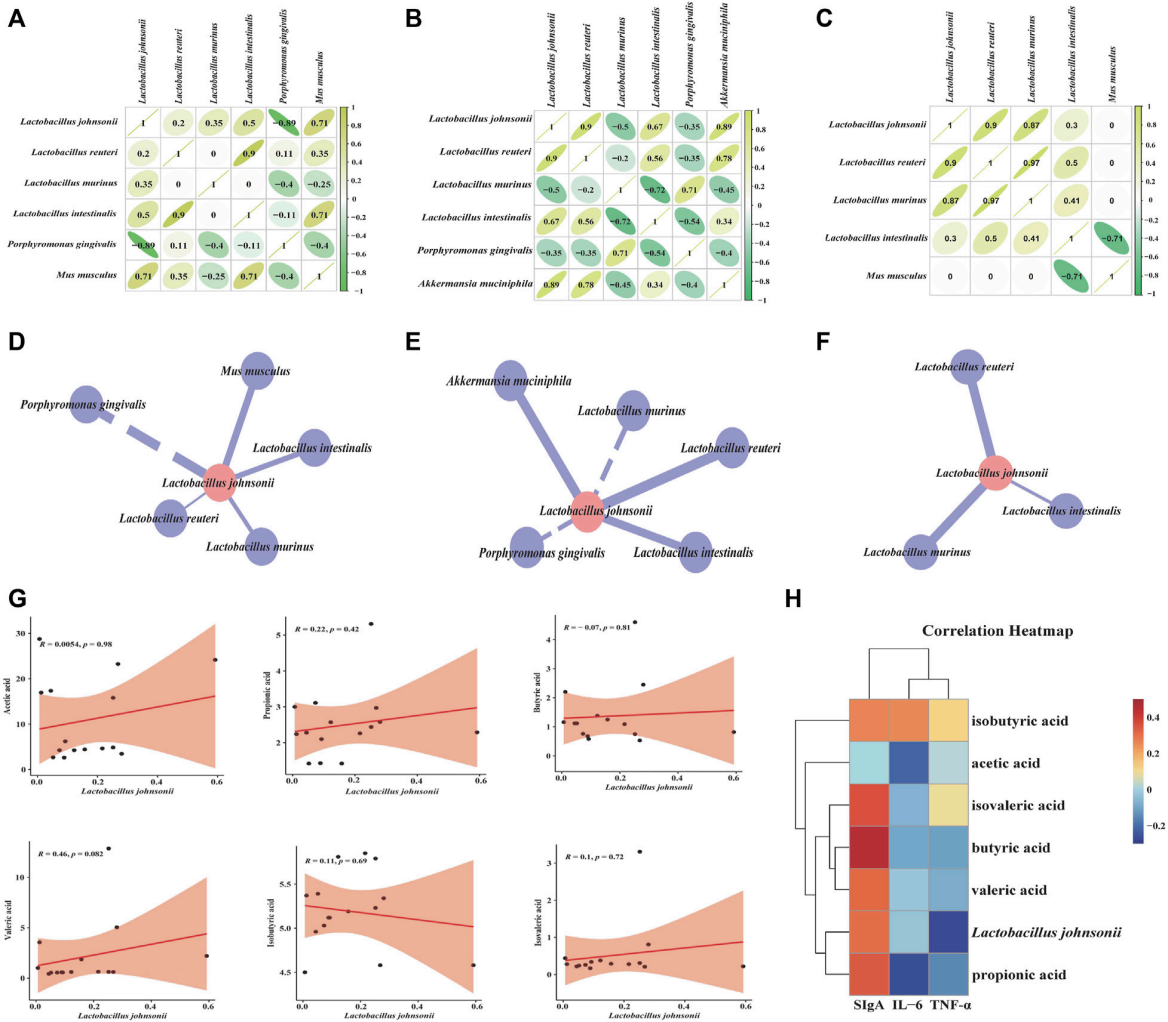
Correlation analysis. **(A)** Correlation coefficient diagram of “characteristic bacteria-characteristic bacteria” in the CZ group, CX group **(B)**, CS group **(C)**. **(D)** Interaction network diagram of “*Lactobacillus johnsonii*-other characteristic bacteria” in the CZ group, CX group **(E)**, CS group **(F)**. **(G)** Scatter plot of *Lactobacillus johnsonii* and SCFAs. **(H)** Correlation heat map between *Lactobacillus johnsonii*, SCFAs and indicators related to gut inflammatory response. CZ, control group (*n* = 5); CX, model group (*n* = 5); CS, SSP group (*n* = 5). IL-6: interleukin-6; TNF-α: tumour necrosis factor-α; sIgA: secretory immunoglobulin **(A)**.

We performed an intra-group correlation analysis and plotted the correlation coefficients of the characteristic bacteria at the species level (Figures 9A–C). The results displayed that the interaction relationship between the characteristic bacteria of mice gut contents was reduced after SSP intervention. Combined with correlation coefficient analysis, we constructed the interaction network between *L. johnsonii* and other characteristic bacteria in the CZ, CX and CS groups, respectively, and explored their interactions before and after modeling and before and after SSP intervention. After modeling, the regulation of *L. johnsonii* by *Akkermansia muciniphila, M. musculus* was changed (Figures 9D, E). The regulation of *L. johnsonii* by *A. muciniphila, Porphyromonas gingivalis, Lactobacillus murinus* was altered, and *Lactobacillus intestinalis* maintained positive regulation of *L. johnsonii* (Figures 9E, F). In a word, we speculated that the changes in the interaction relationship between characteristic bacteria might be due to the effect of SSP on the structure of gut microbiota in diarrhea mice with DYKS.

To further reveal the relationship between characteristic bacteria and SCFAs, correlation scatter plot analysis was performed (Figure 9G). *Lactobacillus johnsonii* was positively correlated with acetic acid, propionic acid, valeric acid, isobutyric acid and isovaleric acid, and negatively correlated with butyric acid. Besides, correlation heatmap analyzed the relationship between the characteristic bacteria, SCFAs and the relevant indicators of gut inflammatory response (Figure 9H). Red squares represented positive correlation, blue squares represented negative correlation, the darker the color the stronger the correlation. Of these, *L. johnsonii* was negatively correlated with IL-6 and TNF-α, and positively correlated with sIgA. Propionic acid, butyric acid and valeric acid were negatively correlated with IL-6 and TNF-α and positively correlated with sIgA. Acetic acid and isobutyric acid were positively correlated with IL-6, TNF-α and sIgA. Isovaleric acid was positively correlated with TNF-α and sIgA, and negatively correlated with IL-6. In short, the interaction of the above factors might be the mechanism of action of SSP in regulating diarrhea with DYKS.

## Discussion

### SSP modulated the gut content microbiota of diarrhea mice with DYKS

In our experiments, we used bioinformatics techniques to analyze the gut content microbiota of mice before and after modeling and drug administration. Diversity analysis identified alterations in the diversity and community structure of the gut contents of the CS group. Characteristic bacteria *L. johnsonii* might act as a biomarker to influence SSP in the treatment of diarrhea with DYKS. Moreover, metabolic function was the functional category that mainly affected the process of SSP in the treatment of diarrhea with DYKS. *Lactobacillus johnsonii* presented strong negative correlations with ko00624 (polycyclic aromatic hydrocarbon degradation) and ko00625 (chloroalkane and chloroalkene degradation) and positive correlations with ko00960. As well, ko00624 (polycyclic aromatic hydrocarbon degradation), ko00625 (chloroalkane and chloroalkene degradation) involved to polycyclic aromatic hydrocarbon degradation and chloroalkane and chloroalkene degradation, which belong to the xenobiotics biodegradation and metabolism in the KEGG secondary classification of metabolic function. ko00960 (tropane, piperidine and pyridine alkaloid biosynthesis) related to piperidine and pyridine alkaloid biosynthesis, which belong to the biosynthesis of other secondary metabolites. Alkaloids are a kind of secondary metabolites widely found in plants and play an important role in resisting biological and abiotic stresses (Xiong et al., 2021). Study highlighted that alkaloids were the main chemical constituents of *E. rutaecarpa* (Juss.) in SSP (Liu et al., 2022). *Euodia rutaecarpa* (Juss.) has modern pharmacological effects such as analgesic, anti-inflammatory, anti-tumour and antioxidant, and has physiological activity on the cardiovascular system, central nervous system, digestive system and reproductive system (Ni et al., 2022). Thus, the interaction between *L. johnsonii* and ko00960 (tropane, piperidine and pyridine alkaloid biosynthesis) might be influenced by the regulation of the drug ingredient of *E. rutaecarpa* (Juss.). On the whole, the intervention of SSP notably altered the structure and function of the gut content microbiota of diarrhea mice with DYKS. Characteristic bacteria *L. johnsonii* might influence SSP for the treatment of diarrhea with DYKS by inhibiting the xenobiotics biodegradation and metabolism and promoting the biosynthesis of other secondary metabolites.

### Close relationship among *Lactobacillus johnsonii*, SCFAs and intestinal inflammatory response

Gut microbiota is a key factor in the health and disease transformation of the organism. They influence the physiological and pathological activities of the organism by metabolizing the nutrients ingested by the body and producing metabolites, mainly SCFAs, which directly or indirectly exchange information with the organism (Tao et al., 2022). Previous study in our group showed that SCFAs in the gut contents of diarrhea mice with DYKS were significantly lower than that of normal mice, and there were correlations between SCFAs and gut content microbiota. Therefore, exploring the interaction between gut microbiota and SCFAs may provide a new direction for the study of disease mechanisms. Combined with the results of this experiment, SSP promoted the propionic acid, butyric acid, isobutyric acid and isovaleric acid in the gut contents of diarrhea mice with DYKS, and characteristic bacteria *L. johnsonii* might play an important role as a biomarker in the treatment of diarrhea with DYKS by SSP. Accumulating evidences revealed that *L. johnsonii* was a widely studied probiotic that colonized a large number of mammals, and its probiotic effects were mainly reflected in a number of aspects such as inhibiting the multiplication of intestinal pathogens, regulating the intestinal microenvironment, enhancing immune function and improving diarrhea (Zhan et al., 2016; Wang et al., 2017; Yang et al., 2019). Yue et al. confirmed that *L. johnsonii* dramatically promoted the acetic acid, propionic acid and isobutyric acid in the faeces of patients with diarrhea caused by enterotoxin-producing *Escherichia coli* (Yue, 2021). Combining the correlation analysis between the characteristic bacteria and SCFAs, we observed positive regulations between *L. johnsonii* and propionic acid, valeric acid, isobutyric acid and isovaleric acid. Taken together, there were correlations between *L. johnsonii* and propionic acid, valeric acid, isobutyric acid and isovaleric acid in the gut contents of diarrhea mice with DYKS after SSP intervention.

Inflammation is a double-edged sword for the health of the body. It is important for the body’s own defence, but excessive or persistent systemic inflammation can have adverse effects (Dao et al., 2016; Rosen and Palm, 2017). TNF-α and IL-6, key cytokines that drive inflammation, could lead to disturbances in the body’s immune regulation and promote inflammation when their expression is increased (Zhang et al., 2011; Zhang et al., 2020b). Also, sIgA is a major effector in the immune response of the gut mucosa, neutralizing pathogens in the gut mucosal epithelium and balancing normal microbiota in the gut (Song et al., 2020). In this study, SSP inhibited the intestinal inflammatory response in diarrhea mice DYKS. Study proved that there was an interaction between gut microbiota and inflammatory response (Al Bander et al., 2020). Some gut microorganisms (e.g., *Lactobacillus johnsonii*) had significant anti-inflammatory effects (Zheng et al., 2021). Cheng et al. evaluated that the relative abundance of *L. johnsonii* in gut contents of ulcerative colitis mice was remarkably lower than that in normal group, the expression of TNF-α mRNA was correspondingly increased, and there was a significant negative correlation between *L. johnsonii* and TNF-α (Chen et al., 2022). Dong et al. emphasized that supplementation of *L. johnsonii* to mothers before delivery and during lactation protected female offspring from experimental colitis (Dong et al., 2022). We also found negative correlations between *L. johnsonii* and TNF-α and IL-6, and a positive correlation with sIgA. Eventually, there was a negative regulatory effect between *L. johnsonii* and intestinal inflammatory response of diarrhea mice with DYKS after SSP intervention.

Propionic acid, butyric acid and valerate have negative regulatory effects on intestinal inflammatory reaction.

**FIGURE 10:**
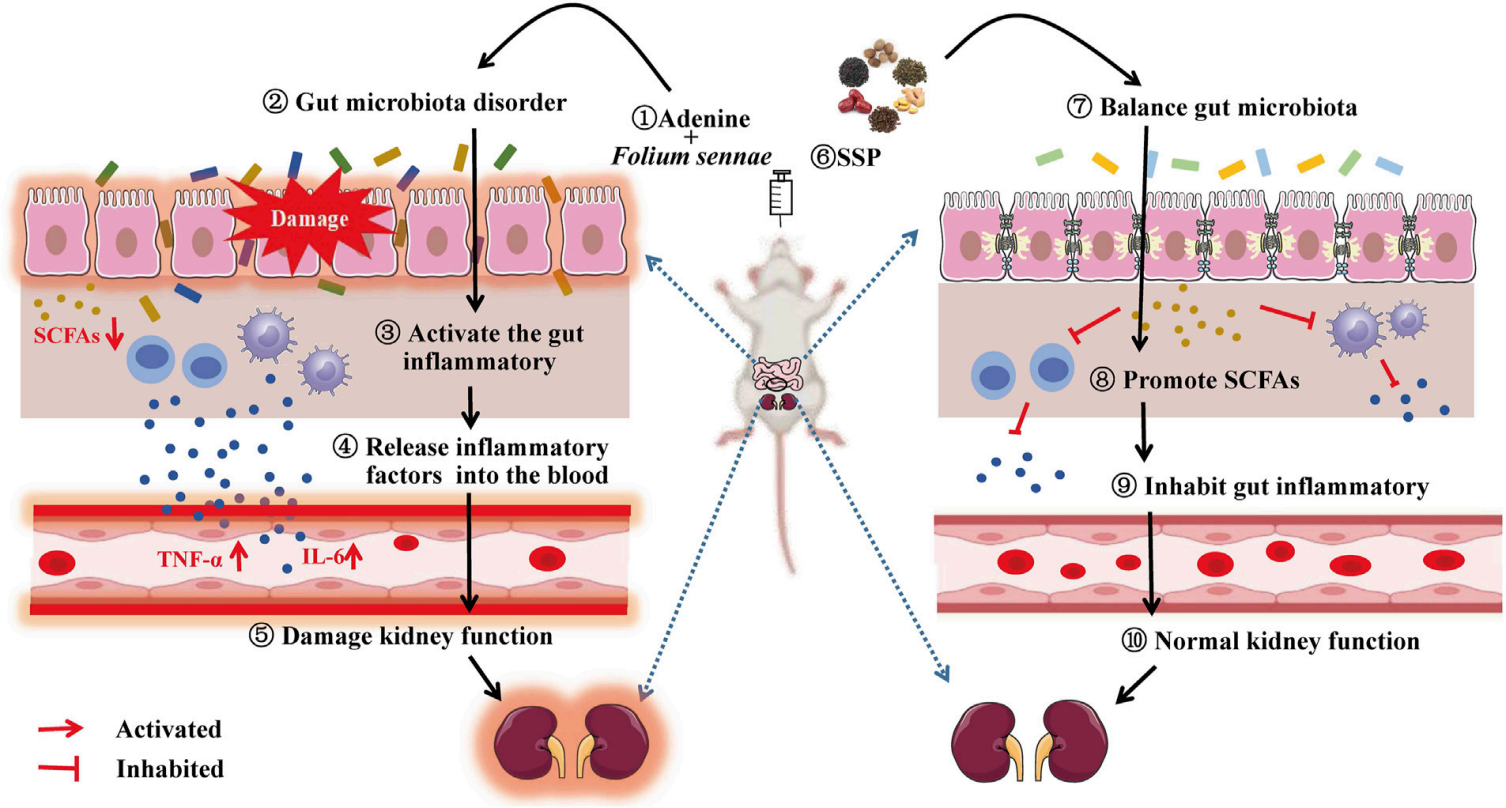
Gut microecological mechanism of SSP in the treatment of diarrhea with DYKS.

Similarly, SCFAs act as signaling molecules that can be involved in the intestinal inflammatory response through different pathways and are key mediators of communication between the gut microbiota and the immune system (Yang et al., 2022). Studies confirmed that SCFAs inhibited the HDACs pathway or activate the GPCR41/43 pathway to suppress macrophage NF-κB-mediated production of pro-inflammatory factors (TNF-α, IL-6, IL-12, etc.) (Al-Lahham and rezaee, 2019); also SCFAs activated GPCRs to upregulate increased IgA secretion and protect gut mucosal barrier function against pathogens (Zhao et al., 2018). Xiang et al. demonstrated that serum levels of TNF-α and IL-6 were significantly reduced in rats with lipopolysaccharide-induced acute respiratory distress syndrome after gavage of a mixture of SCFAs (acetic acid, propionic acid and butyric acid), suggesting that SCFAs could inhibit the expression of inflammatory factors to alleviate acute respiratory distress syndrome (Xiang et al., 2022). Asarat et al. treated IBD mice with butyric acid and found that their plasma IL-6 levels were lower compared to the model group, indicating that butyric acid suppressed the expression of inflammatory factors in the treatment of IBD (Asarat et al., 2016). Luu et al. proved that valerate controlled the function of Th17 by inhibiting the activity of HDAC. In the correlation analysis, propionic acid, butyric acid and valeric acid of diarrhea mice with DYKS were negatively correlated with TNF-α and IL-6, and positively correlated with sIgA (Luu et al., 2019). It could be seen that there were negative regulations between propionic acid, butyric acid and valeric acid and intestinal inflammatory response in diarrhea mice with DYKS after SSP intervention (Figure 10).

## Conclusion

SSP was able to induce changes in the structure and function of the gut content microbiota of diarrhea mice with DYKS, promote the propionic acid, butyric acid, isobutyric acid and isovaleric acid, inhibit the occurrence of gut inflammatory response, and improve the damage to the structure and function of the kidney. In addition, there was positive regulations between *L. johnsonii* and acetic acid, propionic acid, valeric acid, isobutyric acid and isovaleric acid, while negative regulations with intestinal inflammatory response. Propionic acid, butyric acid, valeric acid and gut inflammatory response were negatively regulated. Altogether, we hypothesized that SSP might regulate the expression of inflammatory factors through the “*L. johnsonii*-propionic acid” pathway to suppress the intestinal inflammatory response and achieve the effect of treating diarrhea with DYKS. However, further validation is needed. Considering that the occurrence of diarrhea with DYKS involves multiple factors and the mechanisms involved are complex, and the drug components in SSP may also exert their efficacy mechanisms through multiple targets. Therefore, in the follow-up study, we still need to explore the intervention mechanism of SSP on diarrhea with DYKS through multiple channels.

## Data Availability

The datasets presented in this study can be found in online repositories. The names of the repository/repositories and accession number(s) can be found below: https://www.ncbi.nlm.nih.gov/, PRJNA854690.
